# Biomechanical evaluation of pedicle screw stability after 360-degree turnback from full insertion: effects of screw shape, pilot hole profile and bone density

**DOI:** 10.3389/fbioe.2023.1151627

**Published:** 2023-05-05

**Authors:** Yun-Da Li, Ming-Kai Hsieh, Weng-Pin Chen, De-Mei Lee, Tsung-Ting Tsai, Po-Liang Lai, Ching-Lung Tai

**Affiliations:** ^1^ Department of Biomedical Engineering, Chang Gung University, Taoyuan, Taiwan; ^2^ Department of Orthopaedic Surgery, Spine Section, Bone and Joint Research Center, Chang Gung Memorial Hospital and Chang Gung University College of Medicine, Taoyuan, Taiwan; ^3^ Department of Orthopaedic Surgery, New Taipei Municipal TuCheng Hospital, Built and Operated by Chang Gung Medical Foundation, New TaipeiCity, Taiwan; ^4^ Department of Mechanical Engineering, National Taipei University of Technology, Taipei, Taiwan; ^5^ Department of Mechanical Engineering, Chang Gung University, Taoyuan, Taiwan

**Keywords:** pedicle screws, screw shape, turnback, pilot-hole profile, screw pullout test

## Abstract

Intraoperative pedicle screw depth adjustment after initial insertion, including both forward and backward adjustments, is sometimes necessary to facilitate rod application and ensure that the screw is in the correct position, which is determined by intraoperative fluoroscopy. Adjusting the screw with forward turns has no negative influence on the screw fixation stability; however, screw turnback may weaken the fixation stability. The aim of this study is to evaluate the biomechanical properties of screw turnback and demonstrate the reduction in the fixation stability after the screw is turned 360° from its full insertion position. Commercially available synthetic closed-cell polyurethane foams with three different densities simulating various degrees of bone density were utilized as substitutes for human bone. Two different screw shapes (cylindrical and conical) together with two different pilot hole profiles (cylindrical and conical) were tested. Following specimen preparation, screw pullout tests were conducted using a material test machine. The mean maximal pullout strength between full insertion and 360-degree turnback from full insertion in each setting was statistically analyzed. The mean maximal pullout strength after 360-degree turnback from full insertion was generally lower than that at full insertion. The reduced mean maximal pullout strength after turnback increased with decreasing bone density. Conical screws had significantly lower pullout strength after 360-degree turnback than cylindrical screws. The mean maximal pullout strength was reduced by up to approximately 27% after 360-degree turnback when using a conical screw in a low bone density specimen. Additionally, specimens treated with a conical pilot hole presented a less reduction in pullout strength after screw turnback as compared to those with a cylindrical pilot hole. The strength of our study was that we systematically investigated the effects of various bone densities and screw shapes on screw stability after turnback, which has rarely been reported in the literature. Our study suggests that pedicle screw turnback after full insertion should be reduced in spinal surgeries, particularly procedures that use conical screws in osteoporotic bone. Pedicle screw secured with a conical pilot hole might be beneficial for screw adjustment.

## 1 Introduction

The pedicle screw-rod system is commonly used in various spine surgeries to stabilize vertebrae, correct spine alignment, and accomplish arthrodesis (Gaines, 2000; Lenke et al., 2008; Elder et al., 2015; Hsieh et al., 2020; Li et al., 2021). Despite advances in screw design and surgical instruments in recent decades, pedicle screw loosening is still a frequently discussed issue, with an incidence between 0.8% and 27% (Essens et al., 1993; Wu et al., 2012; Galbusera et al., 2015; Kim et al., 2015). Many predisposing factors may cause screw loosening, and these factors can be divided into patient factors and surgical factors (Galbusera et al., 2015; Shea et al., 2014; Yuan et al., 2021; Wu et al., 2011; Ohba et al., 2019; Bokov et al., 2019). Patient factors include advanced age, comorbidities, and low bone mineral density. Surgical factors include inadequate fixation length, pedicle lateral wall breach, lower axial trajectories, fusion to the sacrum, increased screw pull-out length after rod application, and intraoperative screw turnback.

**FIGURE 1:**
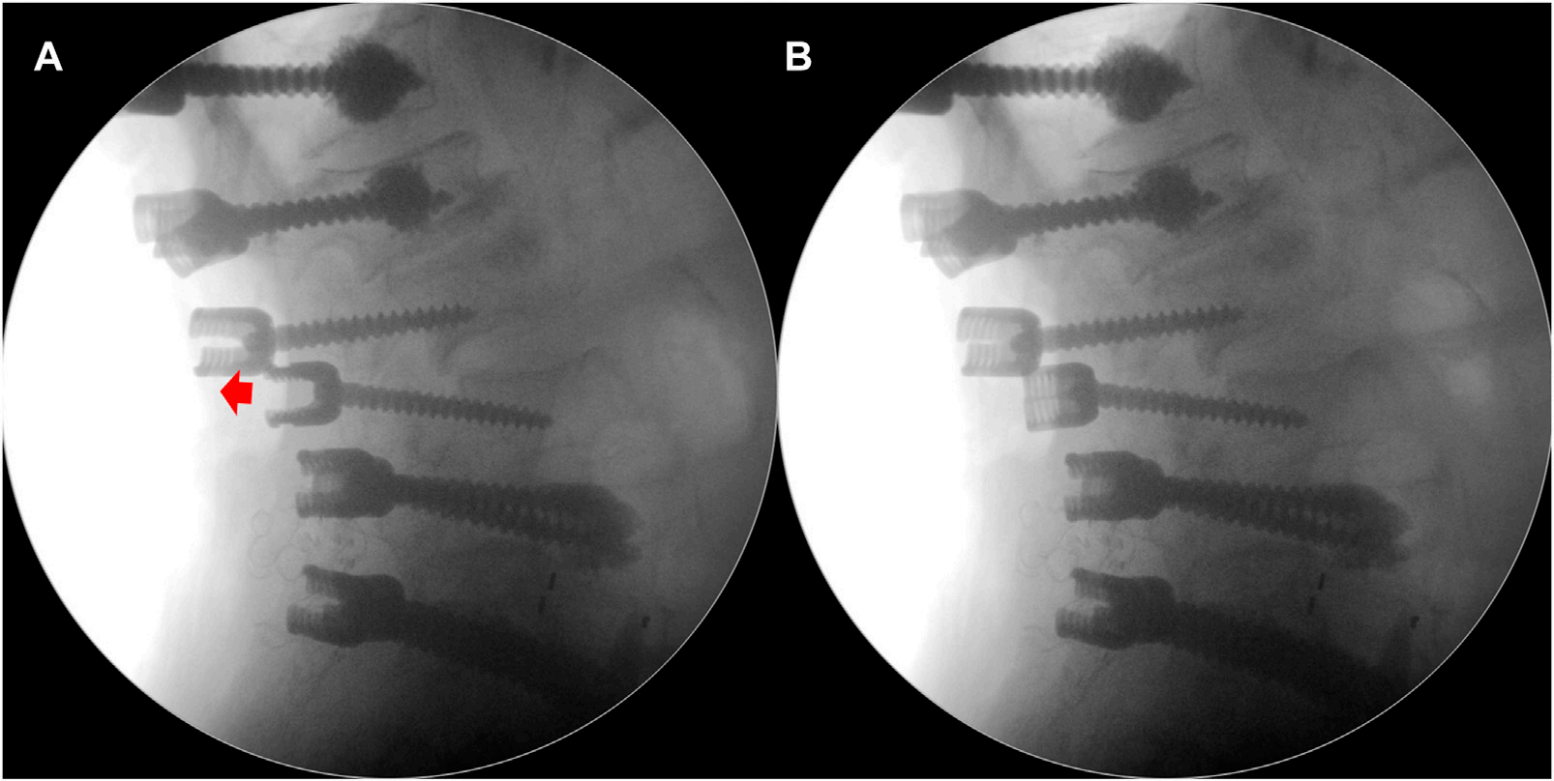
**(A)** One of the lumbar pedicle screws was inserted too far, and it was necessary to adjust this screw backward (red arrow) to facilitate rod application. **(B)** Intraoperative fluoroscope showing the turnback of this screw.

Screw depth adjustment, which involves turning the screw forward or backward through the track after the initial insertion, is sometimes necessary during surgery to facilitate subsequent rod application. In some cases, the screw is inserted too far, exceeding the anterior cortex according to intraoperative fluoroscopy results, and the screw must be turned backward for safety. Screw depth adjustment is usually needed in procedures that require long instrumentation, such as scoliosis surgery, because the rod is difficult to connect to the screws. In this situation, we prioritize inserting screws that could be later adjusted to deeper locations since this method does not have a negative influence on the screw fixation stability. However, there is a limit to how far the screw can be inserted forward, as the tulip of the screw will be blocked by the bony edge of the facet joint. At this point, the only option is to choose another screw and turn it back (Figure 1). Therefore, whether this process negatively affects screw fixation stability and the extent of these effects should be investigated.

According to the literature review, a study (Lill et al., 2000) reported that the pullout strengths of conical screws turned back 180° were notably lower than the cylindrical screws’ pullout strengths in cadaveric spines from 6- to 8-week-old calves. On the other hand, contrasting findings were presented in a separate research (Abshire et al., 2001), which proposed that properly engineered tapered screws have the potential to maintain their pullout strength when rotated up to 180–360° during surgical procedures. This was concluded after conducting paired testing on porcine lumbar vertebrae. Therefore, the impact of adjusting the screw position during surgery on the screw fixation stability remains inconclusive. Another limitation of previous studies is that porcine and calve specimens have denser trabecular matrices than healthy humans. Therefore, these specimens may react to screw turnback differently compared to osteoporotic patients.

Because few studies have systematically explored these effects, we conduct *in vitro* biomechanical research to investigate the difference in the pullout strengths of pedicle screws between full insertion and 360-degree turnback from full insertion using three commercially available test blocks to mimic different degrees of bone quality and two pedicle screws with distinct shapes (cylindrical and conical screws).

## 2 Materials and methods

### 2.1 Foam test block preparation and pilot hole profiles

**FIGURE 2:**
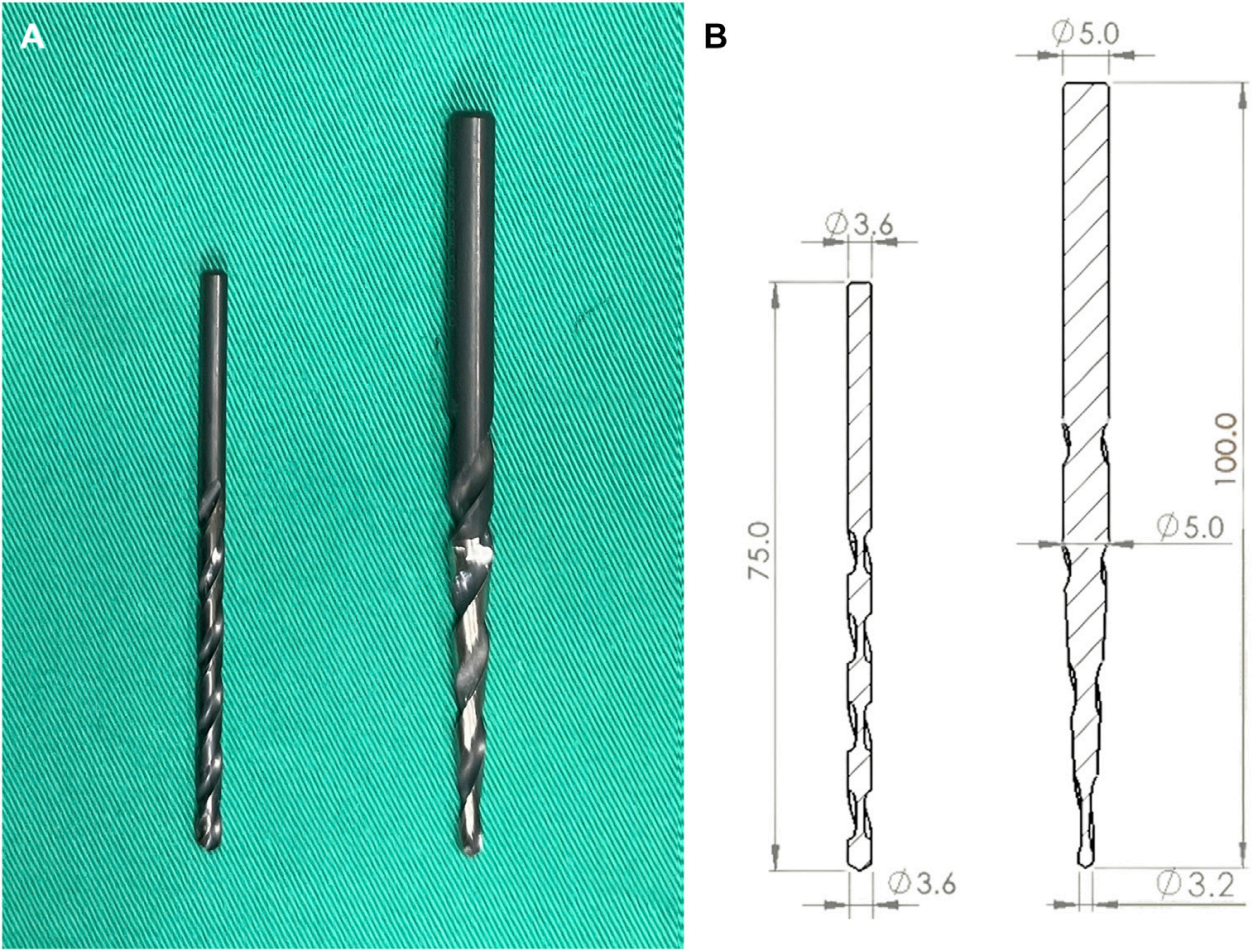
**(A)** Photographs and **(B)** schematic drawings of the two pilot hole profiles, including the cylindrical and conical shapes.

To avoid the influence of differences in morphometry and bone properties among individuals, commercially available synthetic test blocks with three different densities (Pacific Research Laboratory Inc., Vashon Island, WA, United States) that mimic various bone density grades were utilized as experimental substitutes for human bone. (Gibson and Ashby, 1988). Three test blocks made of closed-cell polyurethane foams with densities of 0.12 (7.5 pound per cubic foot, pcf) (Model: #1522-507), 0.24 (15 pcf) (Model: #1522-524), and 0.48 (30 pcf) (Model: #1522-525) g/cm^3^, which simulated cadaveric bones with osteoporosis, healthy bones, and high bone quality, respectively, were chosen (Patel et al., 2008). The foam was cut with a table saw into approximately 50 mm × 50 mm × 80 mm blocks. Two different pilot hole profiles with cylindrical and conical shapes were prepared on the test blocks to simulate the minimally invasive fluoroscopy-guided insertion technique and traditional freehand insertion technique, respectively. The cylindrical and conical shaped pilot holes were created using a cylindrical drill bit with a 3.6 mm diameter and a conical drill bit with a diameter of 3.2 mm at the tip and diameter of 5.0 mm 45 mm from the tip, respectively (Figure 2).

### 2.2 Pedicle screw geometries

**FIGURE 3:**
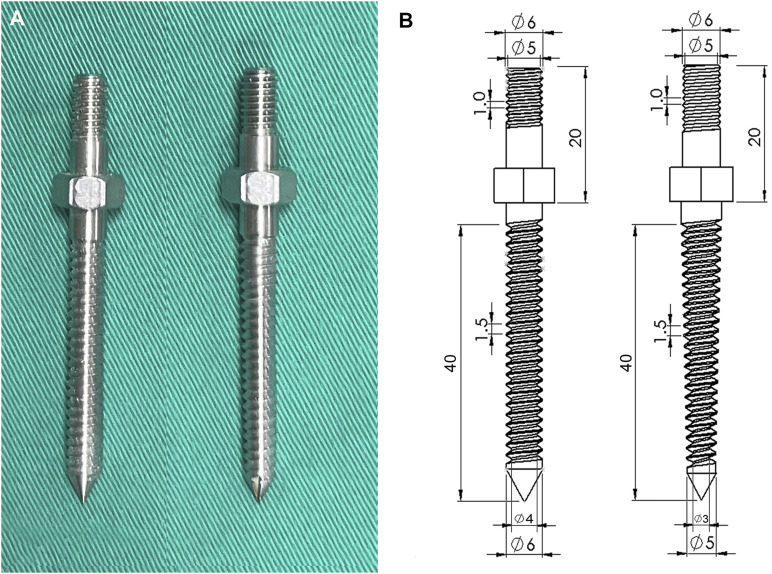
**(A)** Photographs and **(B)** schematic drawings showing the two pedicle screws. The cylindrical screw maintained a constant 6.0 mm diameter from hub to tip; in contrast, the diameter of the conical screw tapered from 6.0 mm at the hub to 5 mm at the tip.

This study employed two types of screws, namely, cylindrical and conical screws. The cylindrical screws had a consistent diameter of 6.0 mm throughout, while the conical screws’ diameter gradually decreased from 6.0 mm at the hub to 5.0 mm at the tip. Both screw designs had a thread depth of 1.0 mm and a thread pitch of 1.5 mm. Moreover, the thread coverage length was standardized at 40 mm, and Figure 3 displays schematic drawings of the pedicle screws.

### 2.3 Experimental groups

**FIGURE 4:**
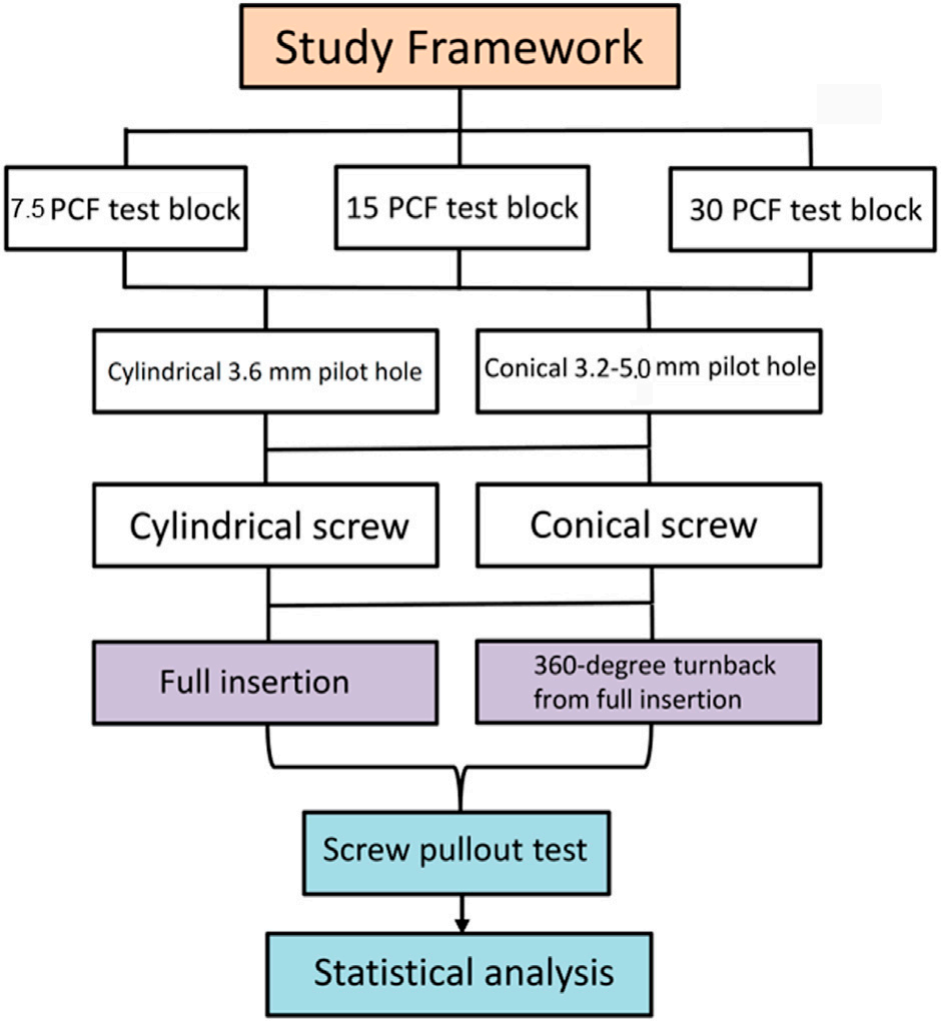
Flowchart of the experimental design.

The experiment was divided into two major parts: full screw insertion and 360-degree turnback from full insertion. First, the pilot hole was created, and the screw was directly inserted into the test block until all the threads were in the block, which was assigned as the full insertion group. The second experimental group was similar to the first group, except the screw was reversed 360° after full insertion. Three test block densities and two screw designs were used in the two groups, and the tests were repeated six times for each setting. The experimental flowchart is shown in Figure 4.

### 2.4 Image analysis and biomechanical pullout testing

**FIGURE 5:**
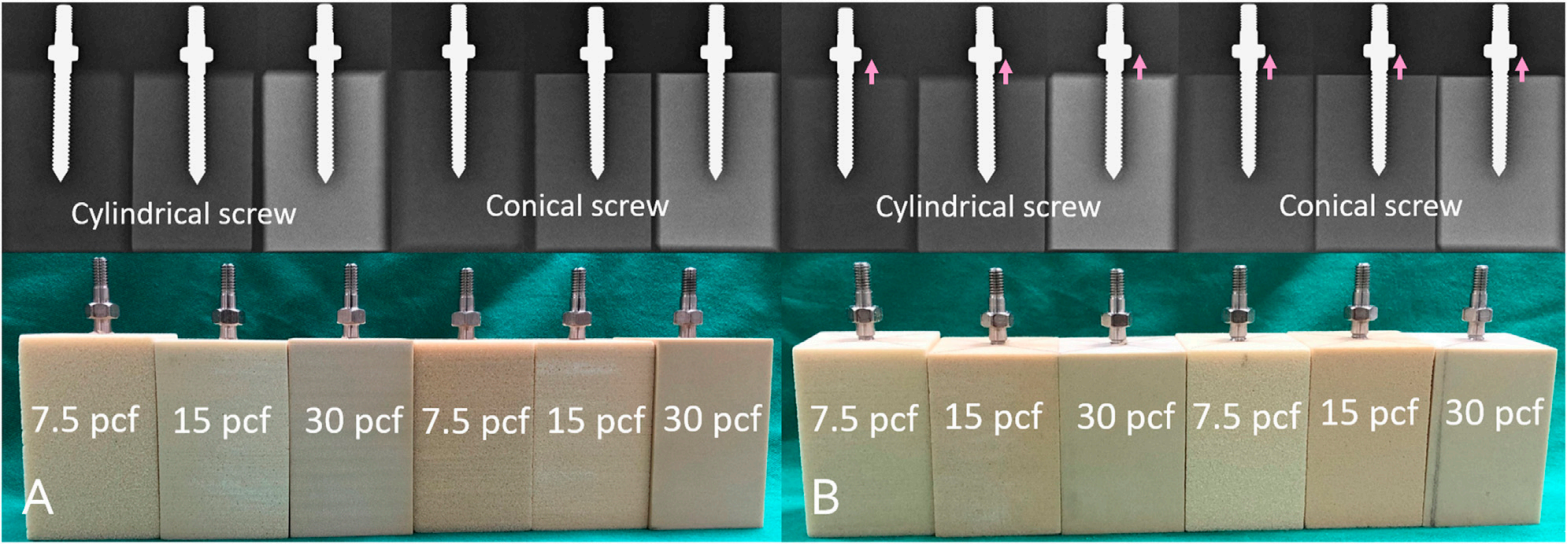
Radiological images and photographs of the specimens in each experimental setting at **(A)** full insertion; and **(B)** 360-degree turnback (pink arrow) from full insertion.

**FIGURE 6:**
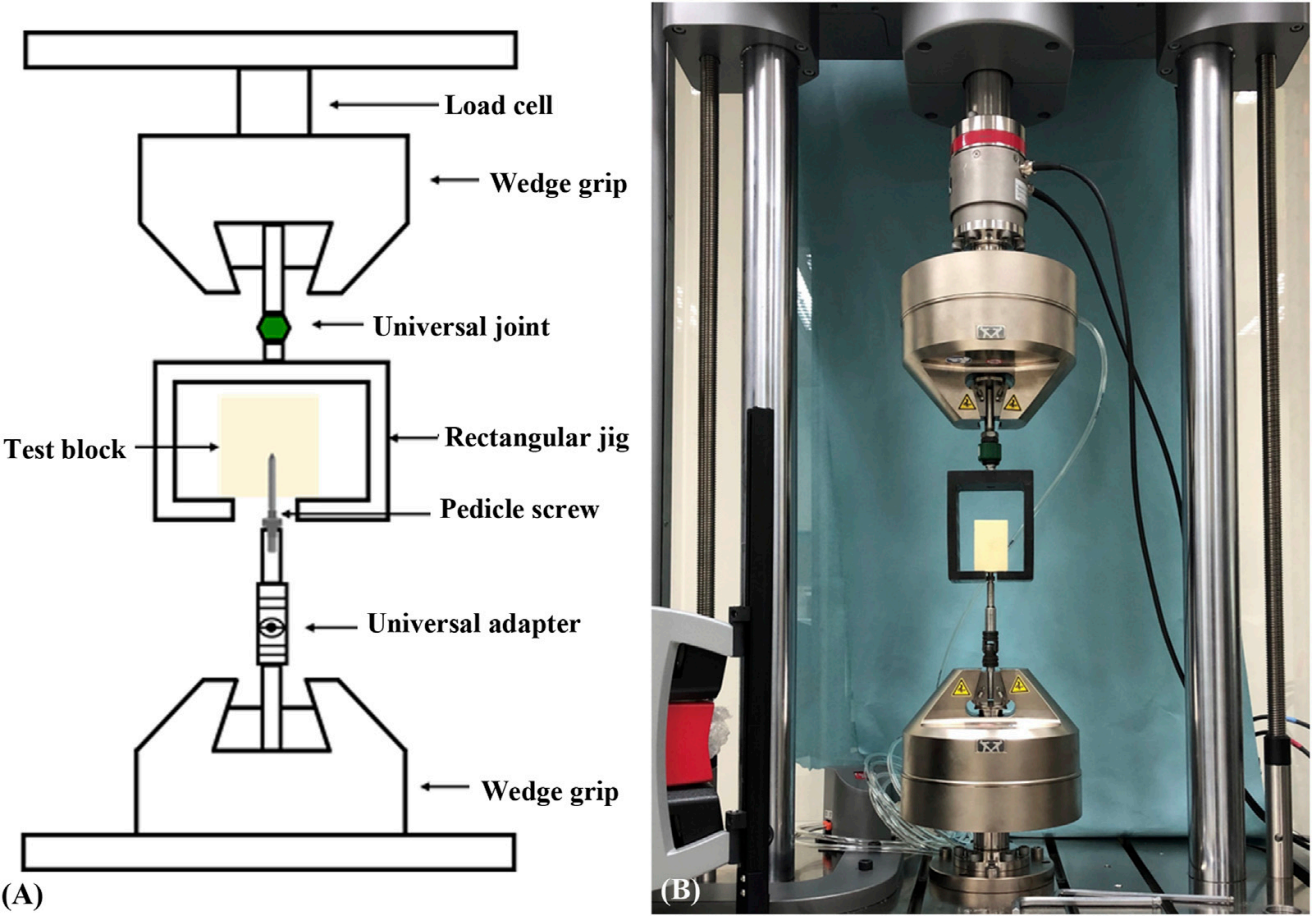
**(A)** Schematic drawing and **(B)** photograph showing the experimental setup of the screw pullout test. The specimen was mounted on a custom-made universal fixture that was capable of self-alignment to ensure that the long axis of the screw was coaxial with the testing machine.

In the two experimental groups, a consistent insertion depth and a trajectory axis that was perpendicular to the insertion plane of the test block were confirmed using X-ray imaging (GE DX300 X-ray machine, Salt Lake City, UT, United States) before pullout testing (Figure 5). The process for the screw biomechanical pullout test was similar to that adopted in our previous studies (Hsieh et al., 2021; Li et al., 2022). After screw insertion, the specimen was affixed onto a specially designed universal fixture that automatically adjusted to align the long axis of the screw with the pullout ram of the testing machine (E10000/E10BMTB19359., Instron Com., Norwood MA, United States). The pedicle screw head was fixed to a 10-mm-diameter rod with an inner thread that matched the outer thread of the screw head. The universal fixture and the rod were then fastened to the upper and lower wedge grips of the Instron testing machine, respectively. The experimental setup for the screw pullout test is depicted in Figure 6. Once the specimen was ready, a constant pullout force of 5 mm/min was applied. The force exerted on the screw during testing was recorded in 0.05-mm intervals until failure. The maximum pullout strength was determined by identifying the peak force recorded during the pullout test for comparison.

### 2.5 Statistical analysis

To assess the impact of screw turnback, the ultimate pullout strengths of the pedicle screws between full insertion and 360-degree turnback from full insertion in each setting were statistically compared. All measurements were expressed as the mean ± standard deviation (SD). Statistical analyses were conducted using the SPSS software (SPSS for Windows version 12.0, SPSS, Inc., Chicago, IL, United States). Differences between groups were assessed using Mann-Whitney U tests, with a significance level of *p* < 0.05.

## 3 Results

### 3.1 Mean maximal pullout strength between full insertion and 360-degree turnback from full insertion of different screw shapes in various bone densities

**FIGURE 7:**
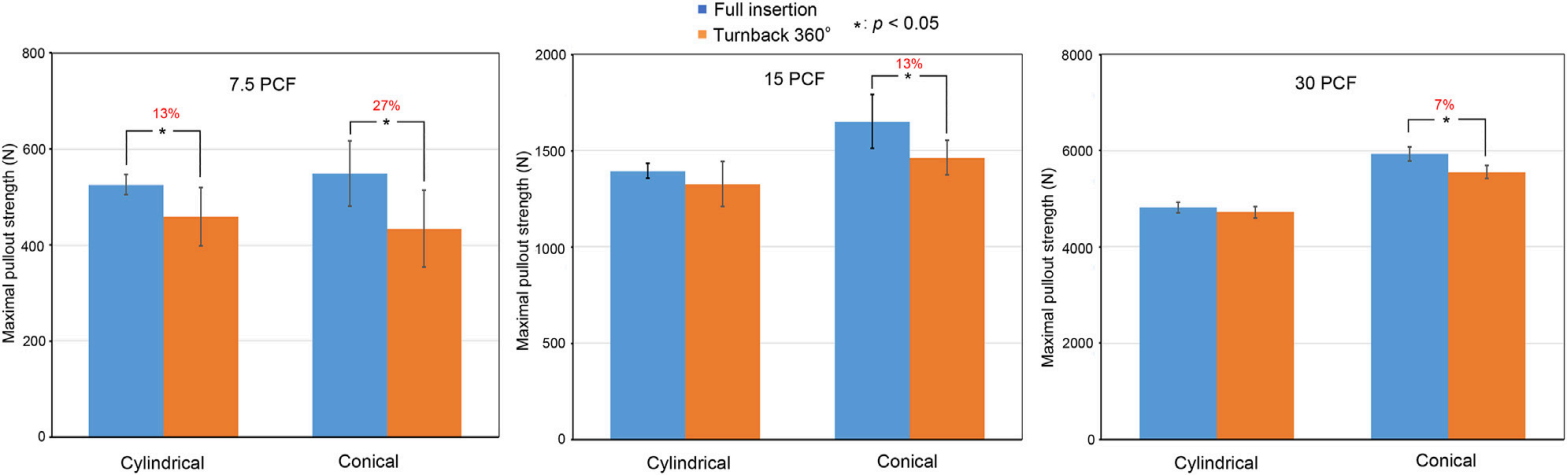
Comparisons of the mean maximal pullout strengths between full insertion and 360-degree turnback from full insertion in the 3.6-mm cylindrical pilot hole using different bone densities (7.5 pcf, 15 pcf, and 30 pcf) and screw shapes (cylindrical and conical). Groups with significant differences are indicated with the “*” symbol and show a reduced percentage.

In the 3.6-mm cylindrical pilot hole group, which simulated the minimally invasive fluoroscopy-guided insertion method, the cylindrical screw at full insertion, cylindrical screw after 360-degree turnback, conical screw at full insertion, and conical screw after 360-degree turnback in the 7.5-pcf group showed mean maximal pullout strengths of 525.19 ± 21.02 N, 458.73 ± 60.02 N, 548.71 ± 67.73 N, and 433.48 ± 79.97 N, respectively, compared with mean maximal pullout strengths of 1396.05 ± 38.15 N, 1325.71 ± 117.22 N, 1652.44 ± 140.93 N, and 1464.42 ± 88.86 N, respectively, in the 15-pcf group and 4822.96 ± 105.87 N, 4717.91 ± 121.77 N, 5936.98 ± 150.43 N, and 5550.35 ± 136.66 N in the 30-pcf group (Figure 7).

**FIGURE 8:**
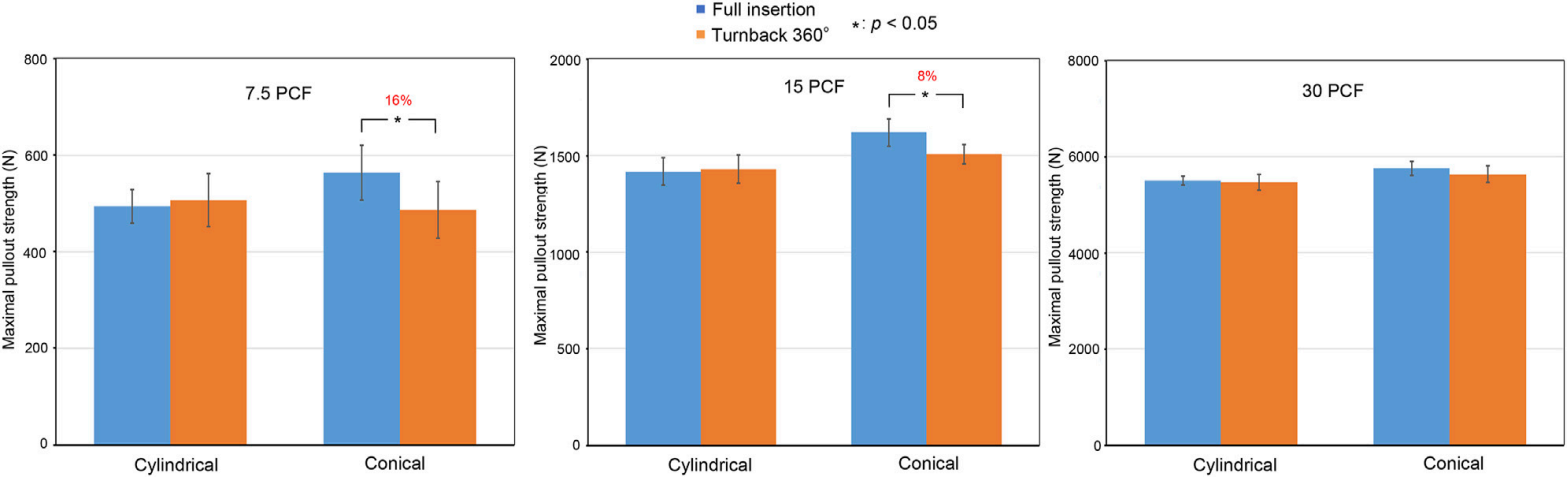
Comparisons of the mean maximal pullout strengths between full insertion and 360-degree turnback from full insertion in the 3.2–5.0 mm conical pilot hole using different bone densities (7.5 pcf, 15 pcf, and 30 pcf) and screw shapes (cylindrical and conical). Groups with significant differences are indicated with the “*” symbol and show a reduced percentage.

In the 3.2- to 5.0-mm conical pilot hole group, which simulated the traditional freehand insertion technique, the mean maximal pullout strengths of the cylindrical screw at full insertion, cylindrical screw after 360-degree turnback, conical screw at full insertion, and conical screw after 360-degree turnback in the 7.5-pcf group were 493.91 ± 34.30 N, 506.55 ± 54.87 N, 563.52 ± 56.67 N, and 487.25 ± 58.42 N, respectively, compared with mean maximal pullout strengths of 1417.33 ± 70.11 N, 1429.86 ± 74.78 N, 1619.65 ± 70.13 N, and 1505.98 ± 50.87 N, respectively, in the 15-pcf group, and 5513.35 ± 90.90 N, 5469.19 ± 162.18 N, 5760.98 ± 143.08 N, and 5640.11 ± 173.84 N in the 30-pcf group (Figure 8).

These data have been organized in Table 1. In both the 3.6-mm cylindrical pilot hole group and the 3.2- to 5.0-mm conical pilot hole group, the mean maximal pullout strength after 360-degree turnback from full insertion was generally lower than that at full insertion.

**TABLE 1:** Mean maximal pullout strength between full insertion and 360-degree turnback from full insertion in each experimental setting.

Pilot hole	Screw shape	Bone density	Mean maximal pullout strength (N)	
			Full insertion	360-Degree turnback
3.6-mm cylindrical pilot hole	Cylindrical screw	7.5-pcf	525.19 ± 21.02	458.73 ± 60.02
		15-pcf	1396.05 ± 38.15	1325.71 ± 117.22
		30-pcf	4822.96 ± 105.87	4717.91 ± 121.77
	Conical screw	7.5-pcf	548.71 ± 67.73	433.48 ± 79.97
		15-pcf	1652.44 ± 140.93	1464.42 ± 88.86
		30-pcf	5936.98 ± 150.43	5550.35 ± 136.66
3.2- to 5.0-mm conical pilot hole	Cylindrical screw	7.5-pcf	493.91 ± 34.30	506.55 ± 54.87
		15-pcf	1417.33 ± 70.11	1429.86 ± 74.78
		30-pcf	5513.35 ± 90.90	5469.19 ± 162.18
	Conical screw	7.5-pcf	563.52 ± 56.67	487.25 ± 58.42
		15-pcf	1619.65 ± 70.13	1505.98 ± 50.87
		30-pcf	5760.98 ± 143.08	5640.11 ± 173.84

### 3.2 Effect of the bone density

In the 3.6-mm cylindrical pilot hole group, the mean maximal pullout strengths in the 7.5-pcf, 15-pcf, and 30-pcf groups were reduced by 27% (*p* = 0.023), 13% (*p* = 0.015), and 7% (*p* < 0.01), respectively, after conical screw turnback. In contrast, the mean maximal pullout strengths were reduced by 13% (*p* = 0.028), 5% (*p* = 0.192), and 2% (*p* = 0.342), respectively, after cylindrical screw turnback. In the 3.2- to 5-mm conical pilot hole group, the mean maximal pullout strengths in the 7.5-pcf, 15-pcf, and 30-pcf groups were reduced by 16% (*p* = 0.023), 8% (*p* = 0.015), and 2% (*p* = 0.213), respectively, after conical screw turnback, while these values were reduced by −2% (*p* = 0.642), −1% (*p* = 0.771), and 2% (*p* = 0.873), respectively, after cylindrical screw turnback. Here, the minus sign indicates an increased value.

The above results indicate a clear trend, namely, that the reduction in the mean maximal pullout strength after 360-degree turnback from full insertion increased with decreasing bone density.

### 3.3 Effect of the screw shape

After full screw insertion, the conical screw obtained a larger mean maximal pullout strength than the cylindrical screw in the 7.5-pcf, 15-pcf, and 30-pcf groups. This result is consistent with previous studies (Lill et al., 2000; Chao et al., 2008; Kim YY et al., 2012; Liu et al., 2020).

After 360-degree turnback from full insertion, in the 3.6-mm cylindrical pilot hole group, the mean maximal pullout strengths after conical screw turnback and cylindrical screw turnback were reduced by 27% (*p* = 0.023) and 13% (*p* = 0.028), respectively, in the 7.5-pcf group. In contrast, the mean maximal pullout strengths were reduced by 13% (*p* = 0.015) and 5% (*p* = 0.192), respectively, in the 15-pcf group and 7% (*p* < 0.01) and 2% (*p* = 0.342), respectively, in the 30-pcf group. In the 3.2- to 5.0-mm conical pilot hole group, the mean maximal pullout strengths after conical screw turnback and cylindrical screw turnback were reduced by 16% (*p* = 0.023) and −2% (*p* = 0.642), respectively, in the 7.5-pcf group, compared with 8% (*p* = 0.015) and −1% (*p* = 0.771) reductions, respectively, in the 15-pcf group and 2% (*p* = 0.213) and 2% (*p* = 0.873) reductions in the 30-pcf group. Here, the minus sign indicates an increased value.

Therefore, we found that the mean maximal pullout strength was reduced more in the conical screw group than in the cylindrical screw group under the same experimental settings after 360-degree turnback from full insertion.

## 4 Discussion

Intraoperative pedicle screw depth adjustment after initial insertion, including both forward and backward adjustment, is sometimes necessary to facilitate rod application and ensure the screw is in the correct position, which is determined by intraoperative fluoroscopy. Adjustment by turning the screw forward has no negative influence on the screw fixation stability; however, screw turnback may weaken the fixation stability. Therefore, we wanted to identify whether screw turnback adversely affected screw fixation quality and determine how much fixation stability was reduced if the screw was reversed 360° from full insertion. This study presents a biomechanical approach to investigate the difference in pullout strength of pedicle screws between full insertion and 360-degree turnback from full insertion using three commercially available test blocks to mimic different degrees of bone quality and two pedicle screw shapes (cylindrical and conical). The strength of our study was that we systematically investigated the effects of various bone densities and screw shapes on screw stability after turnback, an aspect that has received little attention in the available literature. These findings provide spine surgeons with valuable insights into adjusting screw depth during spine surgery.

Numerous studies have demonstrated that utilizing screw pullout testing in a laboratory setting is a dependable approach for assessing the effectiveness of a novel spinal fusion technique or instrumentation (Lill et al., 2000; Abshire et al., 2001; Chao et al., 2008; Kim YY et al., 2012; Amaritsakul et al., 2014; Liu et al., 2020; Hsieh et al., 2021; Li et al., 2022). While the authors acknowledge that clinical screw failures may occur due to various factors, including cyclic loading of screws in multiple planes and the bone’s biological response to the screw over an extended period, axial pullout was chosen to examine screw failure in this study because this method is straightforward and highly reproducible. The results in this study showed that the mean maximal pullout strength after 360-degree turnback from full insertion was generally lower than that after full insertion. Moreover, the reduction in the mean maximal pullout strength after turnback increased with decreasing bone density. Conical screws result in significantly lower pullout strength after 360-degree turnback than cylindrical screws. The results were consistent in the conical and cylindrical pilot hole groups. The mean maximal pullout strength was reduced by up to approximately 27% after 360-degree turnback when using a conical screw in a low bone density specimen.

Several studies have discussed the effects of screw turnback under different circumstances. Lill et al. (Lill et al., 2000) found that the pullout strengths of conical screws turned back 180° were significantly smaller (1.8 kN) than those of cylindrical screws (4.3 kN) using cadaveric spines of 6- to 8-week-old calves. Thus, the authors suggested that pedicle screws, especially conical screws, need to be initially placed at the correct depth and not turned backward. Another study showed completely different results. Abshire et al. (Abshire et al., 2001) conducted a biomechanical analysis comparing the pullout strengths of cylindrical and conical pedicle screws using porcine lumbar vertebrae. Their results showed that conical screws had 17% larger pullout strengths than cylindrical screws (*p* < 0.1) and 50% higher initial stiffness (*p* < 0.05) at full insertion. After the conical and cylindrical screws were turned back 180 or 360° from full insertion, the pullout strengths, stiffness and failure rates remained constant. Three hypothetical mechanisms were proposed, including trabecular bone elastic deformation, slight pedicle expansion, and a specific screw design that held a considerable amount of cancellous bone under compression without crushing it. They concluded that appropriately designed conical screws can be backed out 180–360° for intraoperative adjustment without loss of pullout strength, stiffness or failure. Amaritsakul et al. (Amaritsakul et al., 2014) constructed a biomechanical study to analyze the performance of different screw designs when backed out from full insertion. Their study focused on the influence of different screw designs. Seven conventional pedicle screw designs and one novel design were inserted into 20-pcf polyurethane foam. The results showed that care should be taken when the screws are removed from the full insertion position, particularly cylindrical screws with small thread depths and dual outer core screws.

These studies show that the influence of backward adjustments to the screw position during surgery on the fixation stability remains unknown. One limitation of previous studies is that porcine and calve specimens have denser trabecular matrices than healthy humans, which may influence the screw turnback results. Osterhoff et al. (Osterhoff et al., 2016) reported that the number of trabeculae in the trabecular bone, trabecular thickness and degree of connectivity all affect the mechanical strength of bone. In osteoporosis, these characteristics are all decreased. Therefore, the influence of screw turnback should be more apparent in osteoporotic bone. To the best of our knowledge, no previous studies have compared the effects of screw turnback in bones with different densities. Therefore, we utilized three types of test blocks to mimic different degrees of bone quality in this study.

Other studies have discussed the influence of screw turnback during screw polymethyl methacrylate (PMMA) cement augmentation. Ying et al. (Ying et al., 2012) reported that screw adjustment after cement augmentation, including both forward and backward adjustments, weakened the pullout strength, particularly during forward adjustments. Further advancement of the screw into the solidified cement proved to be detrimental to the bone-cement interface and thus had a significant negative impact on the pullout strength. The results suggested that depth adjustments of pedicle screws should not be performed after cement hardening. In a biomechanical analysis by Chen et al. (Chen et al., 2011), the authors compared the pullout strengths of pedicle screws with cement augmentation at full insertion and after 360-degree turnback from full insertion during the cement hardening process. To evaluate the effect of partial screw removal, the screws were randomly rotated 360° from full insertion 4 min after the introduction of PMMA cement. Typically, PMMA cement does not fully harden until approximately 10–15 min after the powder and liquid are mixed. Therefore, the screw was reversed before the cement was fully hardened in this study. The results showed that the pullout strengths were unchanged (not significant) after partial removal from full insertion. According to these studies and our analysis, the subsequent screw depth adjustment after initial insertion impacts the pullout strength, except in forward screw adjustments without cement augmentation. In other words, screw turnback with or without cement augmentation and forward screw adjustments with cement augmentation all weaken the pullout strength.

Some biomechanical studies have discussed pedicle screw reinsertion using the previous pilot hole and trajectory (Kalemci et al., 2022; Krishnan et al., 2016; Kang et al., 2014). These studies consistently showed that despite a significant reduction in screw insertion torque, there was no significant difference in screw pullout strength with reinsertion using the same screw or a 0.5 mm larger diameter screw through the same trajectory. Therefore, in situations where reversing the pedicle screw is deemed necessary during surgery, particularly in instances of osteoporosis or when utilizing conical screws, it is advised to substitute the screw with a diameter that is 0.5 mm greater to prevent a substantial reduction in pullout strength. This replacement screw should be reinserted along the original path to the appropriate position.

In this study, we found that compared with cylindrical screws, conical screws resulted in significantly decreased pullout strength after 360-degree turnback. This finding can be explained by the geometric configuration of the screw shape. The contact between the conical screws and the surrounding bone became progressively weaker during screw turnback. In the same process, the decrease in the bone contact area is not as apparent with the cylindrical screws. In term of pilot hole profile, our results also demonstrated that, for a given bone density (7.5 pcf, 15 pcf or 30 pcf), specimens treated with a conical pilot hole (3.2- to 5.0-mm) presented a less reduction in pullout strength after screw turnback as compared to those with a cylindrical pilot hole (3.6 mm). This implied that pedicle screw secured with a conical pilot hole might be beneficial for screw adjustment.

The present study had some limitations. First, the test blocks were simply homogeneous material and thus cannot represent real vertebrae conditions. However, the test block was made of uniform polyurethane foam, which reduces the impact of the variability of cadaveric bones and provides an effective and reproducible platform for different degrees of bone mineral density. Therefore, polyurethane foams, with densities typically ranging from 0.16 to 0.64 g/cm^3^, are widely used as standard test materials to mimic human trabecular bone, as stated in ASTM F1839-01 standard specification for rigid polyurethane foam for use as a standard material for testing orthopaedic devices and instruments, published by the American Society for Testing and Materials (ASTM) in 2001 in Pennsylvania. The second limitation was that the cortical bone was not considered in the test block, which results in differences between the experimental pullout force values and clinically determined values. In fact, while the presence of cortical bone would improve the pullout strength, it may not change the comparative results. The average thickness of the pedicular cortical wall in a cadaveric study was 0.6–1.7 mm (Defino and Vendrame, 2007). The screw thread pitch and thread coverage length in this study were 1.5 mm and 40 mm, respectively. The main influence was the overall thread in the homogeneous polyurethane foam. Therefore, the influence of the cortex on 360-degree turnback should be negligible in this experimental design. In addition, only 360-degree turnback was tested. This experimental parameter was chosen because the purpose of this experiment was to investigate the impacts of the screw shape and bone mineral density. Thus, the effects of varying degrees of screw turnback was not explored in this study. Finally, only static screw pullout tests were conducted, neglecting other physiological loading modes. Real-life physiological conditions involve intricate and dynamic loadings at the screw-bone interface, potentially leading to long-term reductions in screw-fixation strength due to fretting and stress shielding effects. Nevertheless, to ensure consistency and reproducibility, all experimental procedures were conducted uniformly. We are confident that our study provides useful information for spine surgeons who must adjust screw depths during spine surgery.

## 5 Conclusion

The mean maximal pullout strength after 360-degree turnback from full insertion was generally lower than that at full insertion. The reduction in the mean maximal pullout strength after turnback increased with decreasing bone density. Conical screws had significantly lower pullout strengths after 360-degree turnback than cylindrical screws. The mean maximal pullout strength was reduced by up to approximately 27% after 360-degree turnback when using a conical screw in a low bone density specimen. Therefore, our study suggests that pedicle screw turnback after full insertion should be reduced in spinal surgeries, particularly when conical screws are used in osteoporotic bone. Pedicle screw secured with a conical pilot hole might be beneficial for screw adjustment.

## Data Availability

The original contributions presented in the study are included in the article/supplementary material further inquiries can be directed to the corresponding authors.
